# The Clinical Value of Flow Cytometric DNA Content Analysis in Patients with Soft Tissue Sarcomas

**DOI:** 10.1080/13577149977604

**Published:** 1999-12

**Authors:** Mustafa Samur, Ali Pamir, Hakan Akbulut, Selim Erekul, Yener Sağlik, Yusuf Yildiz, Dilek Dinçol, Fikri Içli

**Affiliations:** 1 Department of Medical Oncology Ankara University Faculty of Medicine Ibn-i Sina Hospital Sihhiye Ankara 06100 Turkey; 2 Department of Pathology Ankara University Faculty of Medicine Ibn-i Sina Hospital Sihhiye Ankara Turkey; 3 Department of Orthopaedics Ankara University Faculty of Medicine Ibn-i Sina Hospital Sihhiye Ankara Turkey

## Abstract

*Purpose.* The purpose of this study was to evaluate: (1) the correlation between grade and ploidy or S-phase fraction (SPF), (2) the prognostic value of DNA flow cytometric study in soft tissue sarcomas.

*Patients /Methods.* In all, 47 tissue samples from soft tissue sarcoma patients, surgically treated in the same center, were included. Flow cytometric analyses were performed according to a modified version of the original method of Hedley *et al.*

*Results.* DNA ploidy status could be determined in 44 samples out of 47 (success rate 94%). Of these 44, S-phase fraction could be calculated in 34 samples (77%). In the study group as a whole, aneuploidy was significantly correlated with high grade. Survival analyses were carried out in 21 patients with soft tissue sarcoma, all surgically treated in the same center, without chemotherapy or radiotherapy. In univariate analyses, DNA ploidy was found to be a significant factor for overall survival (OAS) and metastasis-free survival MFS. Mean OAS for aneuploid tumors and diploid tumors were 35 and 65 months (*p*=0.034), and mean MFS 23 and 61 months, respectively (*p*=0.005) .

*Discussion.*There is a relation between histological grade and ploidy in soft tissue sarcomas. It appears that low-grade tumors are generally diploid, whereas high-grade tumors tend to be aneuploid. In a subgroup of patients treated only with surgery, DNA ploidy was found to be an important factor for predicting OAS and MFS.


*Sarcoma* (1999) 3, 171–175

ORIGINAL ARTICLE

### The clinical value of flow cytometric DNA content analysis in patients with soft tissue sarcomas

MUSTAFA SAMUR,[1] ALI PAMIR,[1] HAKAN AKBULUT,[1] SELIM EREKUL,[2] YENER SAĞLIK,[3] YUSUF YILDIZ,[3] DILEK DINÇOL[1] & FIKRI IÇLI[1]

[1]*Department of Medical Oncology,* [2]*Department of Pathology,* [3]*Department of Orthopaedics, Ankara University Faculty of Medicine, Ibn-i Sina Hospital, Sihhiye, Ankara, Turkey*

**Abstract**

*Purpose.* The purpose of this study was to evaluate: (1) the correlation between grade and ploidy or S-phase fraction (SPF), (2) the prognostic value of DNA flow cytometric study in soft tissue sarcomas.

*Patients/Methods.* In all, 47 tissue samples from soft tissue sarcoma patients, surgically treated in the same center, were included. Flow cytometric analyses were performed according to a modified version of the original method of Hedley *et al.*

*Results.* DNA ploidy status could be determined in 44 samples out of 47 (success rate 94%). Of these 44, S-phase fraction could be calculated in 34 samples (77%). In the study group as a whole, aneuploidy was significantly correlated with high grade. Survival analyses were carried out in 21 patients with soft tissue sarcoma, all surgically treated in the same center, without chemotherapy or radiotherapy. In univariate analyses, DNA ploidy was found to be a significant factor for overall survival (OAS) and metastasis-free survival MFS. Mean OAS for aneuploid tumors and diploid tumors were 35 and 65 months ($p$=0.034), and mean MFS 23 and 61 months, respectively ($p$=0.005).

*Discussion.* There is a relation between histological grade and ploidy in soft tissue sarcomas. It appears that low-grade tumors are generally diploid, whereas high-grade tumors tend to be aneuploid. In a subgroup of patients treated only with surgery, DNA ploidy was found to be an important factor for predicting OAS and MFS.

**Key words**: *sarcoma, DNA analysis, flow cytometry, prognosis*

### Introduction

Sarcomas are a heterogeneous group of tumors causing diagnostic and therapeutic difficulties.[1,2] The mainstay of therapy is surgery. Adjuvant chemotherapy has been shown to be effective in pediatric rhabdomyosarcoma, osteosarcoma and Ewing's sarcoma but remains controversial in the other adult sarcomas.[3–5] It is important to identify the subset of patients with a poor prognosis because they are candidates for experimental adjuvant chemotherapy regimens in ongoing studies. Unfortunately this selection is not easy and hampered by the heterogeneity of morphology and clinical behavior. Tumor size, histological grade, localization, tumor necrosis, vascular invasion, clinical and pathologic response to neoadjuvant chemotherapy, age and sex, have been all proposed to be prognostic factors for sarcomas.[6–8] Among them, histological grade and tumor size are universally accepted as important factors and most adjuvant trials use a combination of these two factors for patient selection. Unfortunately histological grade of sarcomas has low reproducibility.[9–11]

Flow cytometric analysis of ploidy and S-phase fraction (SPF) has been shown to be useful in prognostication in various tumors.[12] Such studies have also been done for sarcomas and some have found it to be an independent prognostic factor. In some studies it was also shown that flow cytometric analysis could help in grading these tumors.[13–18]

In this study we aimed to evaluate: (1) correlation between grade and ploidy or S-phase fraction, and (2) the prognostic value of DNA flow cytometric study in adult soft tissue sarcomas.

### Patients and methods

### Patients

Patients with histologically proven soft tissue sarcomas between 1989 and 1998 in the Ankara University Faculty of Medicine, Ibni-Sina Hospital, were included in the study. The paraffin-embedded tissue samples of patients were re-evaluated by the same pathologist, for diagnostic verification and grading. Patients with diagnostic and grading disparance were

Correspondence to: Hakan Akbulut, Department of Medical Oncology, Ankara University Faculty of Medicine, Ibn-i Sina Hospital, Sihhiye 06100, Ankara, Turkey. Tel: +90-312-310 33 33 Ext. 21 32; Fax: +90-312-312 16 50; E-mail: hakbulut@dialup.ankara.edu.tr

1357-714X print/1369-1643 online/99/040171-05 © 1999 Taylor & Francis Ltd

**Table 1.** *Patients characteristics*

| Characteristics | *n* (%) |
|---|---|
| Male | 30 (69) |
| Female | 14 (31) |
| Total | 44 |
| Age: median [range] | 41[12–62] |
| Localization | |
| Extremity | 25 (57) |
| Non-extremity | 19 (43) |
| Size | |
| <5 cm | 14 |
| 5–10 cm | 17 |
| >10 cm | 13 |
| Grade | |
| 1 | 11 |
| 2 | 14 |
| 3 | 19 |
| Histological type | |
| Soft tissue sarcoma, not classified | 11 |
| M. Schwannoma | 9 |
| Liposarcoma | 7 |
| Synovial sarcoma | 5 |
| Rhabdomyosarcoma | 4 |
| Leiomyosarcoma | 3 |
| M. fibrous histiocytoma | 3 |
| Other soft tissue tumors | 2 |

excluded. Forty seven tissue samples from patients with soft tissue sarcoma were selected for study. Patient characteristics, treatment and follow up were noted. Patient characteristics are shown in Table 1.

### *Method*

Flow cytometric analyses were performed according to principles of a modified version of the original method of Hedley *et al.*[19] First, histological specimens with Hemotoxylin–eosin were evaluated. From tissues composed of at least 20% malignant cells, 5–6 sections of 50 μm were cut. Areas with tumor necrosis were avoided. The sections were deparaffinized with xylene and rehydrated by changing concentrations of ethanol: 100%, 100%, 95%, 70% and 50%, respectively. After overnight resting in distilled water, the tissue was washed twice with phosphate-buffered saline (PBS). The pieces were digested with 1 mg/ml protease (Sigma Type XXIV) at 37°C for 60 min, shaking by hand every 5–10 min. Following the digestion, 2 ml of cold PBS was added to the solution. The solution was filtered through a 37-μm nylon mesh. After two washings with cold PBS, cell suspensions having $5\times10^5$ to $1\times10^6$ nuclei per ml were prepared with trypsin buffer and centrifuged, then the nuclei were incubated with RNAse solution for 10 min. Finally the nuclear suspensions were stained with propidium iodide and kept in the dark for at least 10 min at 4°C. The samples were then filtered thorough a 37-μm nylon mesh before cytometric analysis.

DNA analyses were carried out with a FACSort flow cytometry (Becton & Dickinson FACSort). Excitation of propidium iodide occurred at 488 nm. At least 20 000 nuclei from each specimen were analyzed. DNA histograms having only one G0/G1 peak with a coefficient of variation (CV) less than 5% were defined as diploid. DNA peaks with CV of more than 10% were not evaluated. The histograms with CV between 5 and 10% were accepted as wide diploid and collected in the same group with diploid ones. The samples were classified as aneuploid if there were at least two distinct Go/G1 peaks, the latter having at least 10% of the total count. Cell cycle analyses were performed by MODFIT software of Becton & Dickinson. DNA histograms having CV of more than 8% were not evaluated for SPF measurements.[20]

### *Statistical analysis*

Correlation between the DNA ploidy and SPF measurements and other parameters were examined using the Pearson test and survivals were calculated by the Kaplan–Meier method. Univariate overall survival (OAS), disease-free survival (DFS) and metastasis-free survival analysis (MFS) were performed using the log-rank test. All calculations were performed using the SPSS 7.0 for Windows statistical package.

### Results

DNA ploidy status could be determined in 44 samples out of 47 (success rate 96%). Of these 44, S-phase fraction could be calculated in 34 samples (77%). Out of 44 soft tissue sarcoma samples, 21 were aneuploid (two were grade 1, five were grade 2 and 14 were grade 3), 23 were diploid (nine were grade 1, nine were grade 2 and five were grade 3). Ploidy status distributions according to grade of all other samples are shown in Table 2.

Correlation between the results of DNA analyses and other parameters are shown in Table 3. Aneuploidy was significantly correlated with high grade.

**Table 2.** *Results of the flow cytometric analyses*

| | Aneuploid | *p* | SPF (mean%) | *p* |
|---|---|---|---|---|
| Grade 1 | 2/11 (lm) | | 7.5 | |
| Grade 2 | 5/14 (lm,lt) | 0.020 | 14.4 | 0.204 |
| Grade 3 | 14/19 (4t) | | 13.2 | |
| Total | 21/44 (48%) | | 11.1 | |

Tetraploid (t) and multiploid (m) samples were given separately under the aneuploid group. SPFS-phase fraction.

**Table 3.** *Correlation between different parameters*

| Factor | Correlation coefficient | *p* |
|---|---|---|
| Grade-ploidy | 0.386 | 0.009* |
| Grade-metastatic presentation | 0.275 | 0.071 |
| Grade-size | −0.090 | 0.569 |
| Ploidy-metastatic presentation | 0.258 | 0.090 |
| Ploidy-size | −0.231 | 0.140 |
| Grade-SPF | 0.247 | 0.159 |
| SPF-metastatic presentation | −0.057 | 0.759 |
| SPF-size | −0.220 | 0.221 |

*Statistically significant.

Tumors with high grade or aneuploid were prone to be metastatic in presentation.

Since sarcoma patients evaluated in this study were histologically heterogeneous and treated with different chemotherapy and radiotherapy protocols, survival analyses were performed only in a subgroup of 21 patients with soft tissue sarcoma, all surgically treated without chemotherapy or radiotherapy. In univariate analyses, DNA ploidy was found to be significant factor for OAS and MFS. Mean OAS for aneuploid tumors and diploid tumors were 35 and 65 months, respectively ($P$=0.034). Mean MFS for aneuploid tumors and diploid tumors were 23 and 61 months, respectively ($P$=0.005; Table 4, Figs 1–2). SPF was a significant factor for DFS. Mean DFS for tumors with SPF≤10% and SPF>10% were 53 and 20 months, respectively ($p$=0.031). Grade was a significant prognostic factor only for DFS. Mean DFS were 52, 19 and 13 months for grade 1, 2 and 3 tumors, respectively ($p$=0.007) (Table 4).

### Discussion

This study shows a correlation of grade and DNA ploidy in sarcomas. Aneuploidy is found to be related both to we find that high grade and metastatic behavior in sarcomas. In keeping with the literature, DNA analysis may predict the aggressiveness of sarcomas.[14] Especially in soft tissue sarcomas, although grade is accepted as the most important prognostic factor, criteria used to determine the grade of a sarcoma are subjective, not standard and poorly reproducible.[9–11] In this respect DNA analyses with flow cytometry may be helpful for assessing the clinical behavior of sarcomas objectively. In this way these tumors can be separated into subsets to define those patients who should or should not be candidates for experimental adjuvant chemotherapy.

To perform a survival analysis, we selected a homogenous group of soft tissue sarcoma composed of 21 patients with localized disease at presentation

**Table 4.** *Survival analyses of 21 soft tissue sarcoma patients treated with surgery only*

| Factor | Not evaluable | OAS | DFS | MFS |
|---|---|---|---|---|
| Ploidy | | | | |
| Diploid | 11 | 65 (58–72) | 36 (18–53)† | 61 (51–70) |
| Aneuploid | 10 | 35 (22–48) | 8 (3–12)† | 23 (11–36) |
| *p* value | | 0.034* | 0.06 | 0.005* |
| Grade | | | | |
| 1 | 9 | 63 (53–73) | 52 (36–67) | 62 (51–74) |
| 2 | 7 | 45 (31–59) | 19 (6–32) | 35 (17–52) |
| 3 | 5 | 45 (28–61) | 13 (5–22) | 29 (9–48) |
| *p* value | | 0.632 | 0.007* | 0.071 |
| SPF | | | | |
| ≤10% | 8 | 62 (51–72) | 53 (35–71) | 57 (42–70) |
| >10% | 7 | 44 (32–56) | 20 (8–31) | 35 (18–52) |
| *p* value | | 0.420 | 0.031* | 0.329 |
| Surgery | | | | |
| Intralesional-marginal | 12 | 46 (34–58) | 9 (3–14)† | 25 (16–33) |
| En-block or radical | 8 | – | 41† | – |
| *p* value | | 0.08 | 0.02* | 0.008* |
| Size | | | | |
| <10 cm | 12 | 53 (45–64) | 25 (2–47)† | 41 (27–54) |
| ≥10 cm | 9 | 60 (44–75) | 35 (7–62)† | 61 (48–75) |
| *p* value | | 0.860 | 0.780 | 0.166 |
| Localization | | | | |
| Extremity | 14 | 55 (47–64) | 25 (8–41)† | 46 (33–57) |
| Non-extremity | 7 | 51 (31–71) | 11 (5–16)† | 46 (27–66) |
| *p* value | | 0.453 | 0.800 | 0.704 |
| Age | | | | |
| ≤40 | 6 | 53 (42–65) | 35 (3–73)† | 45 (27–63) |
| >40 | 15 | 56 (42–72) | 25 (14–35)† | 48 (33–62) |
| *p* value | | 0.887 | 0.952 | 0.829 |

†Median values, all other values are mean (months). *Statistically significant. OAS, overall survival; DFS, disease-free survival; MFS, metastasis-free survival.

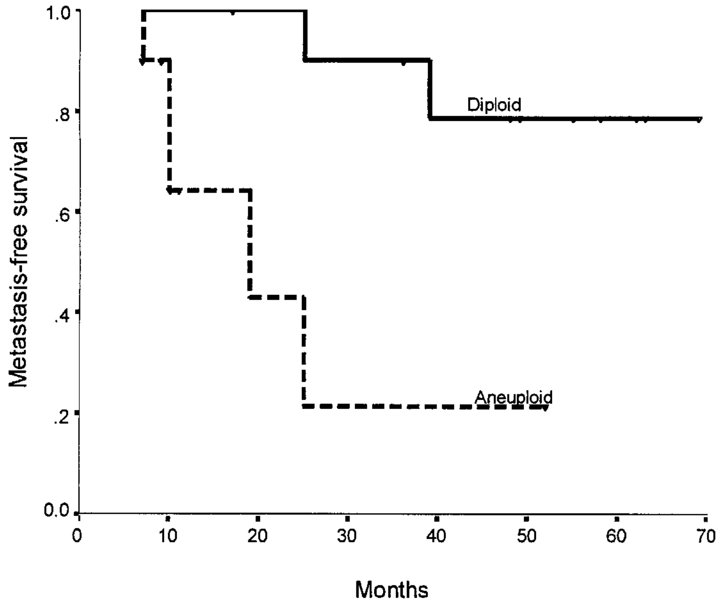

**Figure 1.** *Metastasis-free survival curve of soft tissue sarcoma patients treated with surgery only ($p$=0.005).*

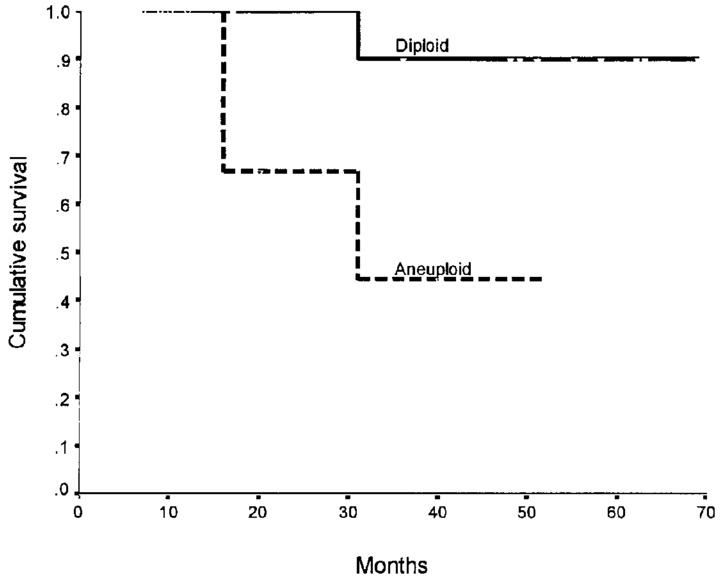

**Figure 2.** *Overall survival curve of soft tissue sarcoma patients treated with surgery only ($p$=0.034).*

and treated only with surgery in the same center without any chemotherapy or radiotherapy. In the analysis of this group, diploid tumors were found to have significantly longer OAS and MFS. SPF, grade and type of surgery were significant prognostic factors for DFS. In evaluating different treatments (such as adjuvant or neoadjuvant chemotherapy) in sarcoma patients, the most important end point should be MFS or OAS, since DFS can be easily affected by the type of surgery, and the high local relapse rate may cause a lower DFS in patients treated with inadequate surgery. In this respect our results are notable, showing that DNA ploidy may be as important as grade of the tumor in evaluation of sarcomas. A similar ploidy–survival relationship is well demonstrated in several other studies.[21–25] Conversely, other studies find SPF a prognostic factor rather than ploidy.[26,27] Rare studies find no corelations in certain type of sarcomas.[28] Conflicting results might be due to the huge variety of histological subtypes of sarcomas or the technical difficulties in flow cytometric DNA analysis of sarcomas. In our study the success rate of 83% in flow cytometric DNA analysis was consistent with others in the literature.[25] Tumor necrosis is the most important factor causing technical difficulties in flow cytometric DNA analysis of sarcomas. Although all samples were re-valuated histologically before study, it is still possible that some samples might be contaminated with necrotic debris and reactive cells.

In conclusion, there is a relationship between histological grade and ploidy in sarcomas. It appears that low-grade tumors are generally diploid, whereas high-grade tumors tend to be aneuploid. At least in the sarcoma group treated with only surgery, we showed that DNA ploidy is an important factor predicting OAS and MFS. With improvement of the technique and standardization with further studies, flow cytometric DNA analysis may provide an objective and reproducible prognostic variable for comparison of series from different centers and possibly for the selection of high-risk patients who may be candidates for adjuvant therapy.

### References

1 Malawer MM, Link MP, Donaldson SS. Sarcomas of bone. In: VT DeVita, S Hellman, SA Rosenberg, eds. *Principles and Practice Of Oncology*, Philadelphia: JB Lippincott, 1997:1789–852.

2 Brennan MF, Casper ES, Harrison LB. Soft tissue sarcoma. In: VT DeVita, S Hellman, SA Rosenberg, eds. *Principles and Practice Of Oncology*. Philadelphia: JB Lippincott, 1997:1738–88.

3 Bruland OS, Phil A. On the current management of osteosarcoma. A critical evaluation and a proposal for a modified treatment strategy. *Eur J Cancer* 1997;33;1725–31.

4 Antman KH. Adjuvant therapy of sarcomas of soft tissue. *Semin Oncol* 1997;24:556–60.

5 Jaffe N, Paed D, Traggis D, *et al.* Improved outlook for Ewing's sarcoma with combination chemotherapy (vincristine, actinomycin D and cyclophosfamide) and radiation therapy. *Cancer* 1976;38:1925–30.

6 Rydholm A. Prognostic factors in soft tissue sarcoma. *Acta Orthop Scand(suppl 273)* 1997;68:148–55.

7 Davis AM, Bell RS, Goodwin PJ. Prognostic factors in osteosarcoma: a critical review. *J Clin Oncol* 1994;12:423–31.

8 Oberlin O, Zucker JM, Brunat-Mentigy M, *et al.* Prognostic factors in treatment of localized Ewing's sarcoma. *Ann Oncol* 1992;3:169.

9 Alvegard TA, Berg NO. Histopathology preview of high-grade soft tissue sarcoma: the Scandinavian Sarcoma Group experience. *J Clin Oncol* 1989;7:1845–51.

10 Hashimoto H, Daimaru Y, Takeshita S, *et al.* Prognostic significance of histologic parameters of soft tissue sarcomas. *Cancer* 1992;70:2816–22.

11 Guillou L, Coindre JM, Banichon F, *et al.* Comparative study of the National Cancer Institute and French Federation of Cancer Centers Sarcoma Group Grading systems in a population of 410 adult patients with soft tissue sarcoma. *J Clin Oncol* 1997;15:350–62.

12 Merkel DE, Mc Guire WL. Ploidi, proliferative activity and prognosis: DNA flow cytometry of solid tumors. *Cancer* 1990;65:1194–205.

13 el Naggar AK, Hurr K, Tu ZN, *et al.* DNA and RNA content analysis by flow cytometry in the pathobiologic assessment of bone tumors. *Cytometry* 1995;19:256–62.

14 Mankin HJ, Connor JF, Schiller AL, *et al.* Grading of bone tumors by analysis of nuclear content using flow cytometry. *J Bone Joint Surg* 1985;3:404–13.

15 Matsuno T, Gebhardt MC, Schiller AL, *et al.* The use of flow cytometry as a diagnostic aid in the management of soft tissue tumors. *J Bone Joint Surg* 1988;5:751–9.

16 Huuhtanen RL, Blomqvist CP, Wiklung TA, *et al.* S-phase fraction of 155 soft tissue sarcomas, correlation with clinical outcome. *Cancer* 1996;77:1815–22.

17 Kreicbergs A, Tribukait B, Willems J, *et al.* DNA flow analysis of soft tissue tumors. *Cancer* 1987;59:128–33.

18 Kroese MCS, Rudgers DA, Wils IS, *et al.* The relevance of the DNA index and proliferation rate in the grading of benign and malignant soft tissue tumors. *Cancer* 1990;65:1782–8.

19 Hedley DW, Friedlander ML, Taylor IW. Method for analysis of cellular content of paraffin-embedded pathological material using flow cytometry. *J Histochem Cytochem* 1983;31:1333–8.

20 Shankey TV, Rabinovitch PS, Bagwell B, *et al.* Guidelines for implementation of clinical DNA cytometry. *Cytometry* 1993;14:472–7.

21 Bauer HC, Kreicbergs A, Tribukait B. DNA content prognostic in soft tissue sarcoma, 102 patients followed for 1–10 years. *Acta Ortop Scand* 1991;62:187–94.

22 El-Naggar A, Ayala AG, Abdul-Karim FW, *et al.* Synovial sarcoma. A DNA flow cytometric study. *Cancer* 1990;65:2295–300.

23 Niggli FK, Powell JE, Parkes SE, *et al.* DNA ploidy and proliferative activity (S-phase) in childhood soft-tissue sarcomas: their value as prognostic indicators. *Br J Cancer* 1994;69:1106–10.

24 Wijnaendts LCD, van der Linden JC, van Diest PJ, *et al.* Prognostic importance of DNA flow cytometric variables in rhabdomyosarcomas. *J Clin Pathol* 1993;46:948–52.

25 Alvegard TA, Berg NO, Baldetorb B, *et al.* Cellular DNA content and prognosis of high grade soft tissue sarcoma: The Scandinavian Sarcoma Group experience. *J Clin Oncol* 1990;8:538–47.

26 Gustafson P, Ferno M, Akerman M, *et al.* Flow cytometric s-phase fraction in soft tissue sarcoma:prognostic importance analysed in 160 patients. *Br J Cancer* 1997;75:94–100.

27 Collin F, Chassevent A, Bonichon F, *et al.* Flow cytometric analysis of 185 soft tissue neoplasms indicates that s-phase fraction is a prognostic factor for sarcomas. *Cancer* 1997;79:2371–9.

28 Fukunaga M, Shimoda T, Nikaido T, *et al.* Soft tissue vascular tumors: a flow cytometric DNA analysis. *Cancer* 1993;71:2233–41.
